# Decoding macrophage heterogeneity in the pulmonary fibrosis lung cancer transition

**DOI:** 10.3389/fimmu.2026.1787094

**Published:** 2026-03-20

**Authors:** Hanming Yu, Mengmeng Zhao, Qiuhong Li, Yuan Zhang

**Affiliations:** Department of Pulmonary and Critical Care Medicine, Shanghai Pulmonary Hospital, School of Medicine, Tongji University, Shanghai, China

**Keywords:** Epigenetic regulation, macrophage heterogeneity, metabolic reprogramming, pulmonary fibrosis-associated lung cancer, tumor microenvironment

## Abstract

Pulmonary fibrosis (PF) significantly increases the risk of lung cancer (LC), but the mechanisms underlying this transition remain unclear. This overview positions macrophage heterogeneity as a central node within the PF-LC continuum. First, we describe important subpopulations of profibrotic and pro-tumor macrophages, including SPP1+, MERTK+, TREM2+, and MARCO+ cells, using high-resolution spatial and single-cell omics technologies. Next, we analyze the fundamental mechanisms that determine their function: the fibrotic microenvironment (e.g., extracellular matrix stiffness, hypoxia) induces profound metabolic reprogramming (e.g., Warburg effect, lipid peroxidation) and stabilizes epigenetic memory (e.g., DNA methylation, histone modifications), locking them into a pathogenic state. This reprogramming occurs through two main pathways: (1) metabolic reprogramming, characterized by aerobic glycolytic conversion and dysregulated lipid metabolism, which stimulates both pathogenic functions and suppression of T cell activity; (2) Epigenetic modifications, including stabilized alterations in DNA methylation, histone modifications, and superactivator patterns, which maintain cells in a tumor-promoting phenotype. As central nodes of communication, these macrophages interact pathologically with fibroblasts and epithelial cells through secreted factors and extracellular vesicles, forming self-reinforcing feedback loops that promote disease progression. We are studying the crucial role of new technologies, particularly multi-omic spatial models and high-precision organoids, in fostering mechanistic discoveries. These discoveries pave the way for new macrophage-focused therapeutic strategies, including the precise stratification of patients using biomarkers from liquid biopsies (such as soluble SPP1 and MARCO) and the development of targeted drug delivery systems for the selective modulation of macrophage function, thus establishing a new paradigm for therapeutic interventions in pulmonary fibrosis with concomitant lung cancer.

## 1 Pulmonary fibrosis to lung cancer as a macrophage-driven disease continuum

Idiopathic pulmonary fibrosis (IPF) is a fatal interstitial lung disease marked by progressive parenchymal scarring and a median survival of only 3–4 years (1). Although anti-fibrotic drugs such as nintedanib and pirfenidone can slow the progression of the disease, they are unable to reverse existing fibrosis or prevent the development of pulmonary fibrosis-associated lung cancer (PF-LC) (2).

IPF confers a markedly elevated lung cancer risk, with a cumulative incidence of 14–30% and a 7- to 20-fold increase compared with the general population (3). PF-LC preferentially emerges within regions of severe fibrotic remodeling and exhibits pronounced resistance to chemoradiotherapy and immune checkpoint inhibitors (ICIs) (4, 5). Importantly, the persistence of high cancer risk even among nonsmokers with IPF (HR = 4.2) implicates the fibrotic microenvironment as an active, disease-intrinsic driver of tumor initiation and treatment resistance (6).

Conventional thinking has primarily focused on dysregulation within the fibroblast activation cycle and epithelial cell damage repair (such as abnormal transforming growth factor-β signaling) (7), yet these mechanisms alone cannot explain the tumor-promoting and immunosuppressive nature of the fibrotic niche. Single-cell omics analyses now demonstrate that IPF progression involves profound immune microenvironment remodeling, resulting in the coexistence of chronic inflammation and deep immunosuppression (8). Elucidating how this unique fibrotic-tumor microenvironment (FTME) reprograms immune cells to forge the molecular link between fibrosis and cancer, thereby shaping the tumor’s distinct biological behavior, remains a central unresolved scientific question.

Macrophages have long been studied through an overly simplified M1/M2 framework, a model that is increasingly insufficient to capture their plasticity and functional diversity in complex pathological contexts (9). In the lung, they are the most abundant innate immune cells, constituting 80% of alveolar immune cells (10), function as central regulators of tissue homeostasis, injury repair, and immune surveillance. The advent of innovative technologies such as single-cell transcriptomics and spatial transcriptomics has enabled the elucidation of their remarkable heterogeneity and the discovery of mechanisms by which they dynamically develop into distinct functional subpopulations in IPF (11).

In PF-LC, new and significantly enlarged macrophage subpopulations can be observed, including subpopulations that are positive for secretory phosphoprotein 1 (SPP1+), positive for myeloid receptor 2 (TREM2+), and positive for macrophage receptor for collagen organization (MARCO+) (12–14). These cell populations collectively create a pro-fibrotic and pro-tumor microenvironment by secreting factors such as TGF-β and PDGF, which drive the fibrotic process, while also releasing pro-tumor mediators such as IL-6 and VEGF (13–16). Essentially, the microenvironment continuously programs this pathological phenotype: extracellular matrix (ECM) signals, localized hypoxia, and metabolic dysregulation continuously reinforce and amplify the profibrotic and immunosuppressive functions of these macrophages through mechanotransduction, HIF-1α signaling, and metabolic reprogramming (17, 18). Previous studies have demonstrated that targeted clearance of CCR2+ monocyte-derived macrophages simultaneously alleviates fibrosis and lung cancer progression, confirming that macrophages represent a highly promising therapeutic target (19).

This review synthesizes macrophage heterogeneity and plasticity across the PF–LC continuum, with a focus on the molecular and microenvironmental mechanisms that drive their pathogenic evolution. We further discuss how fibrotic niches shape macrophage function through metabolic and epigenetic reprogramming and examine the insights provided by emerging spatial multiomic approaches.

## 2 Macrophage heterogeneity and microenvironmental remodeling in PF-LC

Within the fibrotic microenvironment, macrophages derived from tissue-resident macrophages (TRMs) and monocyte-derived macrophages (Mo-Mφ) exhibit marked heterogeneity and plasticity shaped by local cues (20, 21). Distinct macrophage subsets, defined by transcriptional, metabolic, and intercellular interaction programs, collectively drive fibrotic progression and establish a permissive pro-tumorigenic microenvironment.

### 2.1 Formation and functional phenotypes of novel macrophage subsets

The application of high-resolution single-cell and spatial omics technologies to the study of IPF has unveiled multiple macrophage subsets that possess dual pro-fibrotic and/or pro-tumorigenic functions (22, 23).

#### 2.1.1 SPP1+ macrophages

Secretory phosphoprotein 1 (SPP1, also known as osteopontin) and macrophages are present at markedly elevated levels in the lung tissue of patients with idiopathic pulmonary fibrosis and are closely associated with disease severity (22, 24). In fibrotic settings, this subpopulation is primarily defined by its spatial localization and matrix-remodeling functions rather than by tumor-specific immunoregulatory features. This subpopulation is characterized by high expression of SPP1 and MARCO (24, 25). The transcriptional profile of this subpopulation includes potent regulators of the fibrotic process (e.g., lysyl oxidase-like protein 2, type I collagen α-1 chain, type III collagen α-1 chain, COL3A1) as well as several matrix metalloproteinases (e.g., MMP12, MMP19) and pro-fibrotic growth factors (e.g., transforming growth factor β1, TGF-β1; platelet-derived growth factor BB, PDGF-BB) (24, 26).

Spatial transcriptome analysis shows that SPP1+ macrophages are not randomly distributed, but rather accumulate specifically in the central areas of fibrotic lesions and at the fibrotic-carcinogenic interface (27, 28). Within these areas, they form dense cell clusters with α-SMA+ myofibroblasts (α-smooth muscle actin), which together create a local profibrotic microenvironment (27, 28). From a mechanical point of view, SPP1+ macrophages activate the SMAD2/3 and PI3K/AKT signaling pathways of neighboring fibroblasts through the secretion of TGF-β1 and PDGF-BB, which strongly induces their differentiation into highly activated myofibroblasts, ultimately leading to the deposition of extracellular matrix (28, 29). In addition, their highly expressed LOXL2 catalyzes the cross-linking of collagen fibers, increasing the stiffness of the extracellular matrix and resistance to degradation, thus creating a positive feedback loop between mechanical and biochemical factors (30, 31).

While SPP1+ macrophages are primarily characterized in fibrotic lung disease, several of their core features—including extracellular matrix remodeling and close interaction with stromal cells—are conserved across fibrotic and malignant contexts. However, their dominant functional outputs appear context dependent. It is important to note that SPP1+ macrophages also exhibit significant tumorigenic activity (24). SPP1, secreted by this subgroup, can bind to the CD44 receptor on the surface of alveolar epithelial cells, activate the NF-κB signaling pathway, and induce the expression of genes associated with epithelial-mesenchymal transition (EMT), promoting the invasion, proliferation, and angiogenesis of tumor cells (26, 32). *In vivo* studies have shown that specific genetic deletion of SPP1 in macrophages significantly reduces the severity of bleomycin-induced pulmonary fibrosis in mice (32). Clinical cohort studies further confirm the translational potential of SPP1: peripheral blood SPP1 levels in patients with IPF positively correlate with poor prognosis in patients with non-small cell lung cancer (NSCLC) (33, 34). Mack and colleagues obtained consistent results, demonstrating that low plasma SPP1 levels were significantly associated with better clinical outcomes in patients with advanced NSCLC undergoing chemotherapy (35).

Recent studies indicate that SPP1+ macrophages broadly suppress adaptive immunity. SPP1 binds CD44 on T cells, promoting CD8^+^ T cell exhaustion and reducing cytotoxic function, while simultaneously enhancing regulatory T cells (Tregs) expansion and suppressive activity, thereby limiting Th1/Th17 responses and reinforcing immune tolerance (36, 37). High SPP1 signaling also impairs memory T cell function and reduces overall T cell infiltration, contributing to resistance to immune checkpoint inhibitors (38, 39). These effects suggest that SPP1+ macrophages establish a local immunosuppressive niche, linking fibrotic and tumor-promoting processes to adaptive immune dysfunction.

These results suggest that SPP1+ macrophages and their secreted product SPP1 may serve as potential biomarkers for assessing lung cancer risk, while remaining mechanistically distinct from classical tumor-associated macrophages that predominantly regulate antitumor immunity through immune checkpoint pathways.

#### 2.1.2 MERTK+ macrophages

In the pathological microenvironments of fibrosis and cancer, a key role is played by a distinct subpopulation of macrophages, characterized by high expression of the MERTK tyrosine kinase receptor (40, 41). As a critically important phagocytic receptor, MERTK primarily mediates the efficient clearance of apoptotic cells (phagocytosis), a process necessary for resolving inflammation and restoring tissue homeostasis (42). This macrophage subpopulation may also co-express other scavenger receptors (such as CD163) and secreted phosphoproteins (such as SPP1), which together participate in inflammation clearance and tissue repair processes (14, 43, 44).Across fibrotic and malignant settings, MERTK expression marks a macrophage state centered on efferocytosis and tissue remodeling, although the pathological consequences of this program differ substantially depending on the microenvironment.

While MERTK+ macrophages are defined by shared phagocytic and metabolic programs across disease contexts, their dominant functional roles diverge between fibrotic and tumor microenvironments. Transcriptomic analyses of pulmonary fibrosis have shown that MERTK expression is associated with the regulation of certain genes in the glycolytic metabolic pathway (45). Furthermore, studies show that MERTK+ macrophages can enhance phagocytosis by activating the Ucp2/mitochondrial signaling pathway, which influences the course of pulmonary fibrosis (46). However, in the tumor microenvironment, the function of MERTK+ macrophages undergoes significant pathological changes, making them the most important population of immunosuppressive cells (14). The high expression of MERTK in tumor-associated macrophages (TAM) suppresses antitumor immune responses and eliminates immunogenic signals by removing apoptotic cells (47, 48). These immunosuppressive MERTK+ macrophages are known to contribute to tissue remodeling. At the same time, they exhibit strong immunosuppressive and profibrotic potential through the activation of transcription factors such as KLF4 and PPARγ and subsequently secrete cytokines, including interleukin-10 (IL-10) and transforming growth factor-β1 (TGF-β1) (43, 49, 50).

In the tumor microenvironment, MERTK+ macrophages play a central immunosuppressive role by directly inhibiting T cell activity (51). There is compelling evidence that MERTK activation or overexpression is closely correlated with significant upregulation of programmed cell death ligand 1 (PD-L1) in TAMs (52). This MERTK-mediated regulation of PD-L1 allows MERTK+ macrophages to induce CD8+ T cell exhaustion and dysfunction by binding to the PD-1 receptor on the surface of T cells (53, 54). Furthermore, these macrophages can enhance T cell suppression by expressing additional immune regulatory molecules, such as T cell immunoglobulin and mucin domain protein 3 and its ligand galectin-9 (55). Beyond CD8+ T cells, MERTK signaling on macrophages can reduce T cell sensitivity to antigen and lower effector T cell activation at disease sites, thereby limiting responsiveness of both CD4+ and CD8+ T cells to cognate antigen and contributing to immune regulation and tolerance (48, 54). This potent immunosuppressive mechanism mediated by MERTK+ macrophages forms an effective immune barrier in the fibrocarcinogenic microenvironment, contributing to tumor immune evasion.

Studies show that MERTK+ macrophages play a key role in various fibrotic diseases, including pulmonary fibrosis, and contribute to the activation of the ERK-TGFβ1 signaling pathway (46, 56, 57). In the tumor microenvironment, their number is often inversely proportional to the number of tumor-infiltrating lymphocytes (TILs) (58), convincingly confirming their central role in stimulating tumor immune evasion and treatment resistance. Low molecular weight inhibitors targeting MERTK show dual antitumor potential: on the one hand, they directly suppress the growth of several NSCLC subtypes, regardless of motor status (59). On the other hand, they simultaneously modulate the tumor immune microenvironment by acting on TAMs. For example, selective release of UNC2024 into TAMs using specific nanoparticles showed an exceptional antitumor effect in preclinical models (52). This suggests that MERTK inhibition is a promising adjuvant strategy to increase the efficacy of other immunotherapy methods.

#### 2.1.3 TREM2+ macrophages

Within the pathological microenvironments of fibrosis and tumors, TREM2-positive macrophages form another important subpopulation (60–62). As an important pattern recognition receptor, TREM2 recognizes various molecules and associated lipids released during apoptosis or cell damage, thus playing a central role in regulating phagocytosis, metabolism, and inflammatory functions of macrophages (61). Across fibrotic and malignant contexts, TREM2 expression marks a conserved macrophage program centered on lipid sensing, phagocytosis, and metabolic adaptation, although its downstream functional consequences are strongly shaped by the local microenvironment.

In fibrotic lung disease, single-cell sequencing confirms the abundant presence of TREM2+ macrophages in fibrotic foci in patients with idiopathic pulmonary fibrosis (63, 64). Activation of the TREM2 signaling pathway promotes the polarization of M2 macrophages via STAT6 (64), inhibits the apoptosis of alveolar macrophages derived from monocytes, and stimulates the production of profibrotic mediators by macrophages, which promotes the development of pulmonary fibrosis after infiltration into the lungs (60). In a model of bleomycin-induced pulmonary fibrosis, specific elimination of the Trem2 gene in myeloid cells attenuated pulmonary fibrosis by regulating sphingolipid metabolism (59).

In the tumor microenvironment, the tumor effects of TREM2+ macrophages are equally pronounced (52, 60, 62). Within the tumor microenvironment (TME), TREM2+ TAM constitute one of the main immunosuppressive cell populations (60, 62). First, TREM2+ macrophages are recruited into tumor tissue via the CCL2-CCR2 chemotactic axis, where lectin 3 inhibits TREM2-mediated phagocytosis and promotes their conversion into immunosuppressive TAMs, cells that have a significantly reduced capacity for antigen presentation and costimulatory functions *in vivo* (62). Second, TREM2+ TAMs differentiate at different stages into TREM2+/SPP1+ and TREM2+/SPP1− subpopulations, which directly inhibit CD8+ T cell infiltration and cytotoxic function (61, 65). In lung cancer models, the accumulation of TREM2+ TAMs is closely related to resistance to PD-1/PD-L1 immune checkpoint inhibitors (60).

Additionally, TREM2+ macrophages modulate adaptive immunity through mechanisms linked to lipid metabolism and antigen presentation (66–69). High TREM2 signaling skews CD4+ T cell responses toward Tregs and suppresses Th1/Th17 activity, while impairing memory T cell differentiation, thereby promoting local immune tolerance (66). Unlike MERTK+ macrophages, TREM2+ cells limit T cell activation indirectly by modulating metabolic and antigen-presenting pathways rather than primarily through PD-L1 upregulation (68, 70). This contributes to reduced T cell infiltration, functional impairment, and resistance to immune checkpoint inhibitors in tumors.

#### 2.1.4 MARCO+ macrophages

MARCO is a class A scavenger receptor that can recognize and bind various ligands, including modified low-density lipoproteins, bacteria, and components of the extracellular matrix such as collagen (63, 64), and marks a macrophage program observed across both fibrotic and malignant lung microenvironments. In fibrotic lung lesions, MARCO+ macrophages accumulate significantly in areas of active tissue remodeling (64). They are involved in tissue remodeling through endocytosis and degradation of abnormally deposited matrix components. This scavenger-mediated interaction with the extracellular matrix represents a conserved functional feature of MARCO^+^ macrophages across disease contexts. However, in chronic fibrosis, this seemingly homeostatic function may be disrupted, leading to continued pathological remodeling. In addition, MARCO activation modulates macrophage polarization, which normally suppresses excessive inflammation but may shift toward an M2-like profibrotic phenotype in chronic disease conditions (64, 71). A similar situation is observed in lung cancer, where MARCO+TAM weaken the invasive and migratory capacity of tumor cells (60). In several solid tumors, including NMLC, TAMs show high expression of MARCO (72, 73). Unlike PD-L1, MARCO acts as a non-classical immune checkpoint molecule capable of directly suppressing T cell activity (74). When MARCO+ TAM comes into contact with T cells, MARCO directly or indirectly inhibits the proliferation, activation, and cytokine secretion of T cells, forming an effective local immunosuppressive barrier (72). Clinical data show that the density of MARCO+ TAM in lung cancer tissue is significantly correlated with decreased T cell infiltration, poor patient prognosis, and resistance to existing immunotherapies (73). High MARCO signaling promotes expansion of Tregs, suppresses Th1/Th17 CD4+ T cell activity, and impairs memory T cell differentiation, thereby reinforcing local immune tolerance (73, 75). These immunosuppressive effects correlate with reduced infiltration and functional impairment across multiple T cell subsets, contributing to tumor immune evasion and resistance to immune checkpoint inhibitors (76).

**Figure 1:**
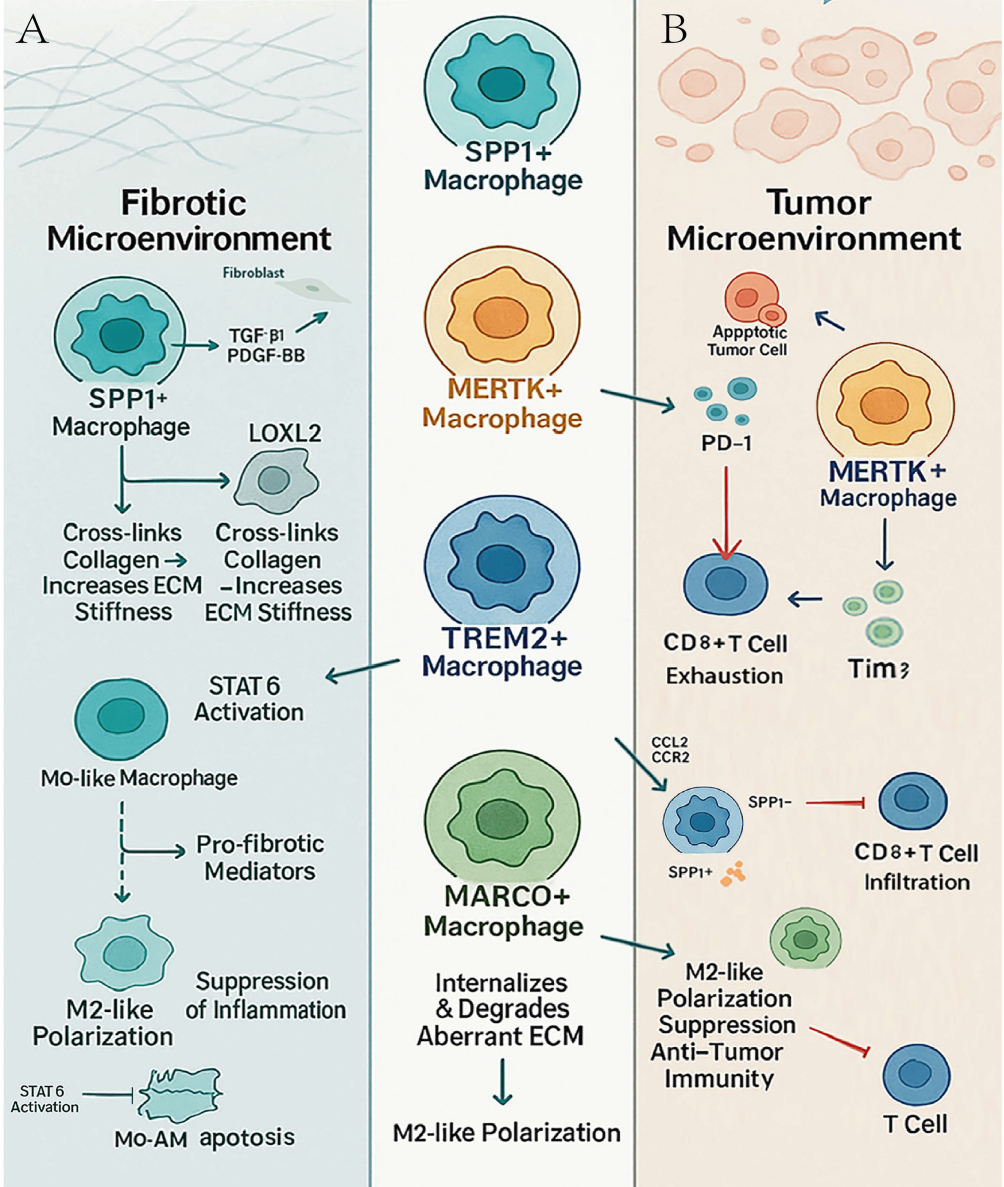
Functional specialization of macrophage subsets across fibrotic and tumor microenvironments. **(A)** Fibrotic microenvironment. **(B)** Tumor microenvironment. SPP1, secreted phosphoprotein 1 (osteopontin); MERTK, MER proto-oncogene tyrosine kinase; TREM2, triggering receptor expressed on myeloid cells 2; MARCO, macrophage receptor with collagenous structure; TGF-β1, transforming growth factor beta 1; PDGF-BB, platelet-derived growth factor BB; LOXL2, lysyl oxidase like 2; ECM, extracellular matrix; STAT6, signal transducer and activator of transcription 6; Mo, monocyte; Mo-AM, monocyte-derived alveolar macrophage; PD-1, programmed cell death protein 1; Tim-3, T cell immunoglobulin and mucin domain-containing protein 3; CCL2, C-C motif chemokine ligand 2; CCR2, C-C motif chemokine receptor 2; CD8^+^, cluster of differentiation 8 positive.

The functional transition of MARCO+ macrophages from pulmonary fibrosis to lung cancer illustrates how a conserved macrophage program acquires context-specific pathological roles. The functional transition of MARCO^+^ macrophages in pulmonary fibrosis and cancer reflects a general pathophysiological continuum. In the early stages of fibrotic lesions, MARCO expression increases as an adaptive response to ongoing tissue damage and destruction of the extracellular matrix, contributing to the removal of waste products and maintenance of tissue homeostasis. Under chronic fibrotic conditions, sustained MARCO expression stabilizes, predisposing macrophages to a state of high MARCO expression. Following malignant transformation, this pre-established phenotype allows MARCO^+^ macrophages to rapidly adopt non-classical immune surveillance functions and suppress T lymphocyte activation and effector responses. Consequently, the fibrotic microenvironment creates the basis for the accumulation of MARCO^+^ macrophages with intrinsic immunosuppressive capabilities, forming the cellular basis for tumor immune evasion (**Figure 1**).

Multiple single-cell studies have reported co-expression of TREM2 and SPP1 within the same macrophage activation state, particularly in immunosuppressive tumor contexts, supporting the notion that these markers delineate overlapping functional programs rather than mutually exclusive subsets (61, 77, 78). By contrast, macrophage populations defined by MERTK or MARCO more often share immunoregulatory features—such as impaired antigen presentation, enhanced efferocytosis, or scavenger receptor activity—without consistent evidence for stable co-expression with TREM2 or SPP1 at the single-cell level (52, 73).

These activation states are further shaped by niche-specific cues, including hypoxia, matrix remodeling, apoptotic burden, and cytokine gradients, and macrophages may transition between them as disease progresses, providing a mechanistic basis for their overlapping yet non-redundant roles in pulmonary fibrosis and lung cancer.

Conceptualizing SPP1+, TREM2+, MERTK+, and MARCO+ macrophages as dynamic states along a functional continuum—rather than isolated subsets—helps reconcile discrepancies across sc-omics studies and highlights the value of integrated spatial and high-dimensional approaches.

#### 2.1.5 Phenotypic and functional features of scar-associated macrophages

Scar-associated macrophages (SAMacs) represent a distinct macrophage state within fibrotic lung tissue, originally defined by high expression of CD9 and TREM2 and further characterized by co-expression of SPP1 (79, 80). Although SAMacs partially overlap transcriptionally with SPP1^+^ and TREM2^+^ macrophage subsets discussed above, they are distinguished by their stable localization within fibrotic niches, rather than by marker expression alone.

A key feature that differentiates SAMacs from other profibrotic macrophage populations is their spatial restriction to fibroblastic foci, where active extracellular matrix deposition occurs (14, 80). In contrast, broadly defined SPP1^+^ macrophages are distributed more diffusely within injured interstitial regions, while MARCO^+^ macrophages predominantly reside in the alveolar space and function in surfactant clearance and debris scavenging (71). SAMacs are uniquely positioned in close proximity to α-SMA^+^ myofibroblasts, enabling sustained interactions within localized fibrotic microenvironments (81).

Functionally, SAMacs differ from other profibrotic and tumor-associated macrophage subsets by integrating structural and immunomodulatory roles. Whereas MERTK^+^ macrophages or classical tumor-associated macrophages primarily exert their effects through soluble immunosuppressive or pro-fibrotic mediators (46, 52), SAMac-enriched niches are associated with dense extracellular matrix architectures that restrict immune cell infiltration and shape a permissive tissue microenvironment (82). In PF-LC, this ECM-centered regulatory mode differentiates SAMacs from SPP1^+^ or TREM2^+^ tumor-associated macrophages that predominantly influence antitumor immunity via cytokine and immune checkpoint signaling (52, 79, 83), supporting the view that SAMacs represent a fibrotic niche–adapted macrophage state distinct from other subsets described here.

#### 2.1.6 Spatiotemporal dynamics and clinical translational value of macrophage subsets

Within the pathological continuum ranging from pulmonary fibrosis to lung cancer, macrophage subpopulations are not static entities but exhibit pronounced spatiotemporal dynamics and functional plasticity, determined both by their cellular origin and by signals from their microenvironment. Analysis of pseudotemporal trajectories and other studies show that these macrophages (especially Mo-Mφ) predominantly differentiate into profibrotic phenotypes in fibrotic lesions, with the SPP1+ subpopulation being a typical example. They directly stimulate tissue remodeling and matrix deposition through the secretion of mediators such as SPP1 and TGF-β1 (28, 29). At the same time, subpopulations such as MERTK+, TREM2+, and MARCO+ collectively support the profibrotic microenvironment by mediating uncontrolled phagocytic elimination, lipid reprogramming, and abnormal matrix elimination.

With the onset of malignant transformation, the functional axis of these pre-existing macrophage subpopulations undergoes a decisive shift toward strong immunosuppression. MERTK+ and TREM2+ macrophages suppress T-cell function through the positive regulation of molecules such as PD-L1 (52, 60); MARCO+ macrophages directly inhibit T cells as non-classical checkpoints (72); while SPP1+ macrophages promote tumor invasion through mechanisms such as the induction of epithelial-mesenchymal transition, in addition to sustained profibrotic activity (29). This functional shift from “fibrogenesis” to “tumorigenesis and immunosuppression” creates a robust immune-privileged barrier at the interface between fibrosis and carcinogenesis, thereby laying the cellular foundation for tumor development and progression.

This spatiotemporal evolution underscores substantial translational potential. Targeting macrophage-associated pathways such as MERTK or MARCO may confer dual benefits by attenuating fibrotic progression while alleviating tumor-associated immunosuppression. In parallel, dynamic shifts in macrophage populations provide a molecular basis for non-invasive biomarker development; notably, a set of 42 shared immune-related pathogenic genes between IPF and lung cancer has been used to construct a risk stratification model for cancer development in IPF patients (83). Integrating multi-omics data to establish multimodal biomarker panels capturing macrophage subset–specific features may therefore enable early detection, refined risk stratification, and personalized therapeutic guidance across the PF–LC continuum.

#### 2.1.7 Spatial and developmental context of macrophage remodeling

Macrophage remodeling along the PF–LC continuum is a multistage, spatially organized process rather than a phenomenon confined to a single anatomical compartment. Chronic injury, aging, and systemic inflammation bias bone marrow hematopoiesis toward myeloid lineages, generating circulating monocytes with altered inflammatory and metabolic potential (84, 85). Although partial transcriptional conditioning may occur in the circulation, systemic cues alone are insufficient to establish stable macrophage phenotypes in the absence of tissue-specific signals (86, 87).

Lineage-tracing and single-cell studies consistently identify the fibrotic lung as the dominant instructive niche in which macrophage identity is defined and maintained (10, 88, 89). Within this microenvironment, hypoxia, extracellular matrix remodeling, cytokine gradients, and apoptotic cell burden impose sustained metabolic and functional constraints that stabilize disease-associated macrophage states (88, 90, 91). Accordingly, macrophage populations such as SPP1^+^, TREM2^+^, MERTK^+^, and MARCO^+^ macrophages are best understood as lung niche–dependent activation states rather than pre-determined lineages (15, 91, 92).

Collectively, this hierarchical model integrates systemic myeloid bias with tissue-dominant reprogramming and provides a unified framework for macrophage heterogeneity across the PF–LC axis (10, 85).

### 2.2 Dynamic regulation of macrophage phenotype by the fibrotic microenvironment

The fibrotic microenvironment of the lung comprises a dynamic network of physical, chemical, and biological signals that constantly reprogram the phenotype and function of macrophages, thereby reinforcing the self-perpetuating cycle of fibrosis and carcinogenesis. This section highlights how key factors in the microenvironment —mechanical stress, hypoxia, and pulmonary dysbiosis—lead, through various molecular and metabolic pathways, to a polarization of macrophages toward profibrotic and immunosuppressive states, ultimately creating a niche conducive to the development and progression of PF-LC.

#### 2.2.1 Matrix stiffness

Mechanical forces within IPF lesions have a significant impact on the development and progression of lung cancer (93). Pathological reorganization of the extracellular matrix within fibrotic lesions leads to a significant increase in matrix stiffness, which in turn alters the expression of profibrotic genes (94). This increased matrix stiffness profoundly affects the functional phenotypes of macrophages, including their polarization state, through mechanotransduction pathways (95, 96). The matrix metalloproteinase/thromboxane/integrin axis is an important signaling pathway mediating stiffness-induced profibrotic phenotypes of macrophages (97). Macrophages perceive the mechanical properties of the ECM mainly through surface integrins such as αvβ3. Upon contact, macrophages immediately induce an increase in cytoplasmic calcium concentration in fibroblasts, leading to the translocation of the transcription coactivators NFATc1 (T-cell-activated factor 1) and YAP (Yes-associated protein) transcription coactivators, ultimately promoting fibroblast activation within a few hours (98).

The transcription coactivators YAP and TAZ (transcription coactivator with PDZ domains) are key components of this mechanotransduction pathway (99, 100). In high-stiffness substrates, the integrin-Src/FAK signaling axis stimulates the transfer of YAP/TAZ from the cytoplasm to the cell nucleus (100). In the cell nucleus, YAP/TAZ binds to transcription factors of the TEAD family and induces the expression of profibrotic genes such as TGFB1 and ACTA2. This, in turn, increases the ability of macrophages to secrete TGF-β1, which promotes the differentiation of neighboring fibroblasts into myofibroblasts (101, 102). *In vitro* studies show that highly rigid matrices (16 kPa) increase M2 macrophage polarization and LOXL2 expression (103). These data are confirmed in animal models: specific elimination of YAP/TAZ in macrophages significantly reduces bleomycin-induced pulmonary fibrosis, underscoring the key role of mechanical signals in macrophage-mediated fibrosis (104). Furthermore, a three-dimensional model of co-cultured lung adenocarcinoma cells and macrophages, created using a network of interpenetrating hydrogels, has further demonstrated the decisive influence of macrophage phenotype and extracellular matrix stiffness in inducing EMT (105).

#### 2.2.2 HIF-1α couples fibrotic and tumor-promoting macrophage programs

In the fibrous microenvironment, hypoxia acts as a decisive ecological signal that modifies macrophage function through the central regulatory factor HIF-1α (106). Under hypoxic stress conditions, the inhibition of prolyl hydroxylases (PHD) prevents the degradation of HIF-1α, promoting its stability and transport to the nucleus (107). Upon reaching the cell nucleus, HIF-1α initiates two pathological programs by binding to the promoters of target genes.This transcriptional reprogramming stimulates the expression of factors that have both profibrotic and oncogenic effects, including SPP1, vascular endothelial growth factor A (VEGFA), and platelet-derived growth factor (PDGF) (107–109). For example, SPP1 not only induces profibrotic differentiation of macrophages through NF-κB activation, but also directly stimulates collagen synthesis in adjacent fibroblasts (110). At the same time, VEGFA contributes to tumor expansion by stimulating neovascularization (manifested as increased microvascular density) (111), while PDGF-BB-induced autocrine chains increase MCP-1/CCL2 levels and attract additional macrophages to reinforce the microenvironment that favors tumor growth (112).

In addition to directly regulating transcription, HIF-1α influences this pathological phenotype by triggering metabolic changes. It activates important glycolytic enzymes, such as pyruvate kinase M2 (PKM2) and lactate dehydrogenase A (LDHA) (113, 114), increasing glycolysis and promoting lactate accumulation, a characteristic metabolic state closely related to the proinflammatory and profibrotic functions of macrophages (115–117). This metabolic reprogramming is not an isolated phenomenon; HIF-1α also synergistically regulates histone-modifying enzymes, influencing chromatin availability and enhancing the expression of its protumoral gene signature (118, 119). Its key role has been confirmed pharmacologically: HIF-1α inhibition significantly reduces the expression of profibrotic mediators and improves pathological progression in pulmonary fibrosis and lung cancer (120–122), confirming that HIF-1α is a key regulator of abnormal macrophage activation.

#### 2.2.3 TLR4–NF-κB–dependent macrophage programming in pulmonary dysbiosis

Recent studies show that lung dysbiosis acts as an important regulatory factor and alters macrophage phenotypes in the fibrotic microenvironment (123). The lung microbiota of patients with idiopathic pulmonary fibrosis undergoes significant changes in its composition, characterized by a noticeable proliferation of bacteria such as Bacteroidetes (e.g., Legionella), which replace the homeostatic dominance of Firmicutes and Actinobacteria (124). This dysbiotic state exacerbates the burden on the lungs from bacterial endotoxins, particularly lipopolysaccharides (LPS). As a canonical ligand of Toll-like receptor 4 (TLR4), LPS causes prolonged activation of MyD88-dependent NF-κB signaling cascades in macrophages, which trigger persistent pro-inflammatory and pro-tumorigenic programs (125, 126).

**Figure 2:**
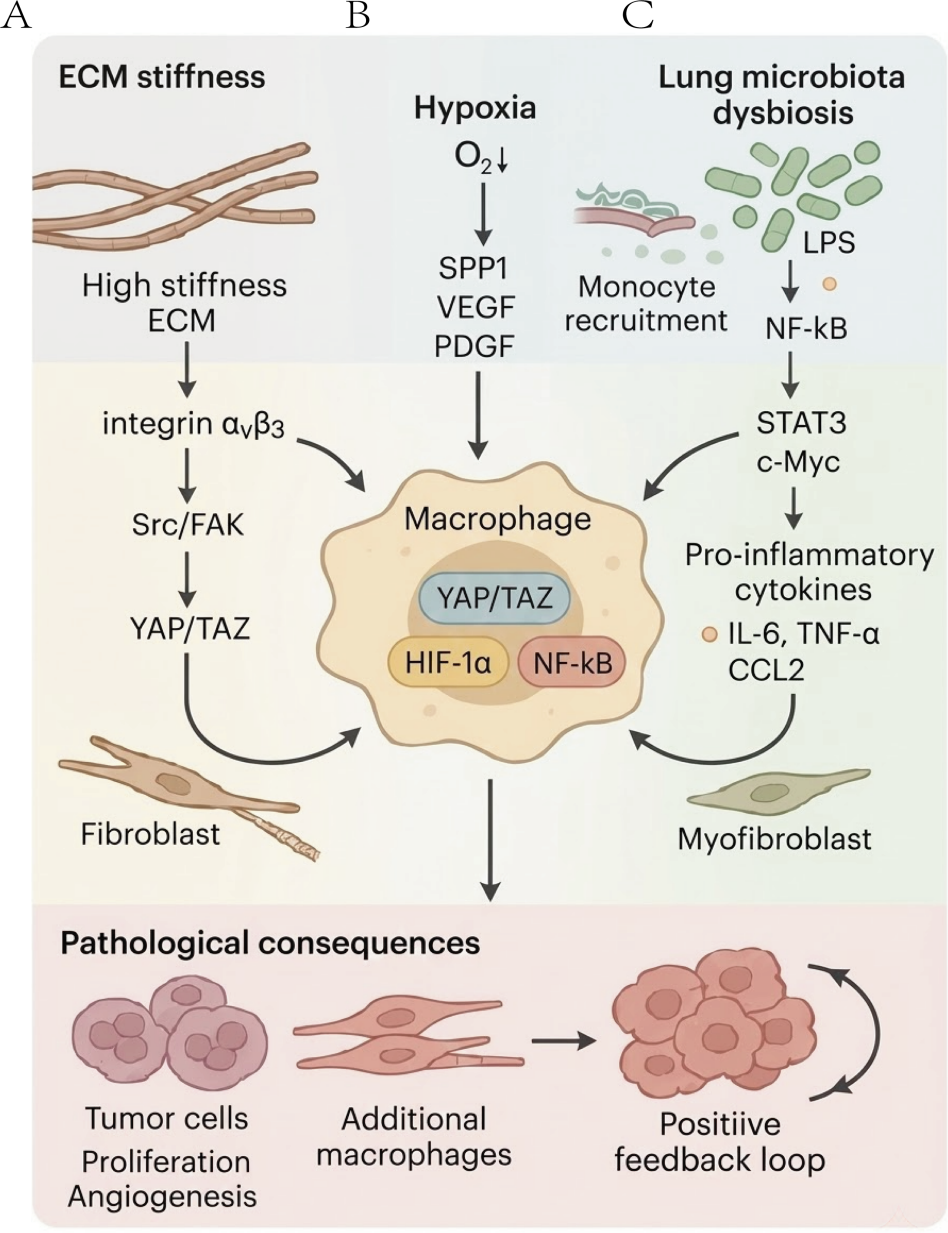
Dynamic regulation of macrophage phenotypes by the fibrotic lung microenvironment. **(A)** ECM stiffness. **(B)** Hypoxia. **(C)** Lung microbiota dysbiosis. ECM, extracellular matrix; αvβ3, integrin alpha V beta 3; Src, Src proto-oncogene non-receptor tyrosine kinase; FAK, focal adhesion kinase; YAP, yes-associated protein; TAZ, transcriptional coactivator with PDZ-binding motif; HIF-1α, hypoxia inducible factor-1 alpha; VEGF, vascular endothelial growth factor; PDGF, platelet-derived growth factor; LPS, lipopolysaccharide; NF-κB, nuclear factor kappa-light-chain-enhancer of activated B cells; STAT3, signal transducer and activator of transcription 3; c-Myc, MYC proto-oncogene, bHLH transcription factor; IL-6, interleukin 6; TNF-α, tumor necrosis factor alpha.

Activation of the TLR4-NF-κB pathway in macrophages induces the release of proinflammatory cytokines and chemokines, including IL-6, TNF-α, and CCL2. This promotes the polarization of resident macrophages and recruits circulating monocytes to the fibrotic microenvironment via CCR2-mediated signaling (127, 128). *In vitro* experiments confirm that lipopolysaccharide stimulation rapidly induces the secretion of IL-6 and the profibrotic cytokine TGF-β1 by macrophages (129). *In vivo* studies substantially confirm these findings: pharmacological or genetic inhibition of the TLR4 signaling pathway significantly attenuates the severity of bleomycin-induced pulmonary fibrosis (130).Furthermore, the pathological consequences of TLR4-NF-κB activation extend beyond acute inflammation and lead to the formation of a persistent pro-tumor microenvironment. Chronic TLR4 signaling regulates important oncogenic transcription factors, such as STAT3 and c-Myc, which amplify proliferative and anti-apoptotic signals transmitted by macrophages to nearby epithelial or tumor cells (131, 132). At the same time, LPS-stimulated macrophages release mutagenic factors, such as reactive oxygen species (ROS) and nitric oxide (NO), which cause DNA damage and promote malignant transformation (133, 134). The clinical importance of this signaling pathway is confirmed by the observation of significant TLR4 upregulation in the tissues of patients with IPF and concomitant lung cancer (135), positioning the microbiota-controlled TLR4 signaling pathway as a critical mechanism in the continuum of inflammation, fibrosis, and carcinogenesis (**Figure 2**).

#### 2.2.4 Genetic drivers of macrophage dysfunction in PF–LC

Genetic predisposition critically shapes the pulmonary microenvironment in which macrophage remodeling occurs and contributes to inter-individual variability in fibrosis progression and lung cancer risk (136, 137).The MUC5B promoter polymorphism (rs35705950), a well-established germline risk factor for idiopathic pulmonary fibrosis, alters epithelial barrier function, mucociliary clearance, and chronic injury patterns, thereby indirectly conditioning macrophage niches rather than directly determining macrophage identity (138–140).Notably, this variant also predicts poorer overall survival in non–small cell lung cancer patients receiving definitive radiotherapy, supporting a role in long-term tissue remodeling and disease trajectory across fibrotic and malignant contexts (138).

In parallel, age-associated clonal hematopoiesis of indeterminate potential (CHIP), driven by somatic mutations in genes such as TET2 and DNMT3A, biases myeloid cells toward heightened inflammatory and profibrotic programs (141, 142).Experimental and clinical studies indicate that CHIP-derived myeloid cells amplify cytokine production and macrophage-mediated tissue damage under chronic inflammatory stress, linking systemic somatic mutations to local macrophage behavior in diseased lungs (143, 144).

Together with environmental factors such as pulmonary dysbiosis and persistent TLR4–NF-κB signaling, germline susceptibility and CHIP likely determine the magnitude and persistence of macrophage activation, providing a mechanistic explanation for heterogeneous PF–LC progression among patients exposed to similar injurious stimuli (145, 146).

#### 2.2.5 Fibrosis and dormant cell reactivation

Beyond promoting local malignant transformation, emerging evidence suggests that fibrotic lung microenvironments may also influence metastatic seeding and tumor cell dormancy (147, 148). Pulmonary fibrosis is characterized by excessive extracellular matrix deposition, increased tissue stiffness, hypoxia, and vascular remodeling, features that have been implicated in regulating tumor cell survival and immune evasion in lung cancer–associated niches (149, 150). These fibrosis-associated alterations partially overlap with microenvironmental programs described in pre-metastatic or pro-dormancy niches within the lung (151).

Macrophages within fibrotic lung tissue contribute to these processes by shaping matrix organization, angiogenic balance, and local immune surveillance (152, 153). In lung cancer specifically, macrophage-mediated immunosuppression is reinforced by metabolic pathways, including purine metabolism, which sustain T cell dysfunction and may facilitate survival of dormant or newly seeded tumor cells (154, 155). Although direct clinical evidence linking pulmonary fibrosis to metastatic dormancy regulation in PF–LC remains limited, these observations support a conceptual framework in which fibrosis-associated macrophage niches may influence metastatic behavior beyond their direct effects on primary tumor growth.

## 3 Regulatory networks underlying macrophage function

### 3.1 Metabolic reprogramming

Signals in the fibrous microenvironment, including hypoxia, cytokines, and matrix stiffness, trigger metabolic reprogramming of macrophages, commonly characterized by a transition from oxidative phosphorylation to Warburg-type glycolysis (156). Importantly, this metabolic adaptation is not uniform across all macrophages but varies according to local microenvironmental cues and functional states. In addition to promoting cellular adaptation, metabolites obtained through glycolysis also act as signal mediators that contribute to creating an immunosuppressive microenvironment and provide energy for tumor growth (156).

#### 3.1.1 Lactate metabolism

Persistent hypoxia and inflammation within the fibrotic foci activate a cascade of HIF-1α signals within the macrophages (106), leading to an upregulation of glycolytic enzymes such as hexokinase 2 (HK2) and LDHA. This ultimately leads to an accumulation of lactate concentrations that are many times higher than those found in healthy tissue (114). The lactate secreted by macrophages subsequently has a pro-tumor effect (157, 158). First, it creates an immunosuppressive barrier by acidifying the microenvironment and directly inducing apoptosis, thereby disrupting the cytotoxic mechanisms of CD8+ T lymphocytes and natural killer (NK) cells (159, 160). Second, lactate enhances the immunosuppressive and pro-tumor activity of pathological macrophages by activating their receptor ligand GPR81 (161, 162). Finally, a high-lactate environment exacerbates tissue acidosis by altering the structure of the local matrix and the functional state of cells, thereby accelerating tumor progression (163).

SPP1+ macrophages preferentially localize to hypoxic regions, exhibiting enhanced glycolysis and elevated SLC2A1/GLUT1, which drives lactate production and M2-like polarization (164). Co-localization with cancer stem cells creates metabolic niches where lactate promotes EMT and tumor invasion, functioning as both a hypoxic adaptation and a signaling hub that sustains immunosuppression (165, 166). Although direct experimental evidence in fibrosis-to-tumor transition is limited, these observations suggest that SPP1^+^ macrophages may similarly sustain immunosuppressive, pro-invasive niches in fibrotic regions, providing a testable hypothesis for future studies.

#### 3.1.2 Dysregulated lipid metabolism

In the fibrotic microenvironment, macrophages engulf significant amounts of oxidized lipids and apoptotic debris via scavenger receptors such as CD36, leading to extensive accumulation of intracellular lipid droplets (LDs) and the formation of a characteristic foamy phenotype (167–169). This state of lipid overload represents a critical functional dichotomy (168). On the one hand, the accumulation of polyunsaturated fatty acids makes cells highly vulnerable to lipid peroxidation and ferroptosis, especially in the case of dysfunction of the antioxidant enzyme glutathione peroxidase 4 (GPX4) (170). However, profibrotic macrophages achieve adaptations critical for their survival by increasing GPX4 expression and thus acquiring stable resistance to ferroptosis, which allows them to maintain their viability and sustain profibrotic function in the long term (171).

**Figure 3:**
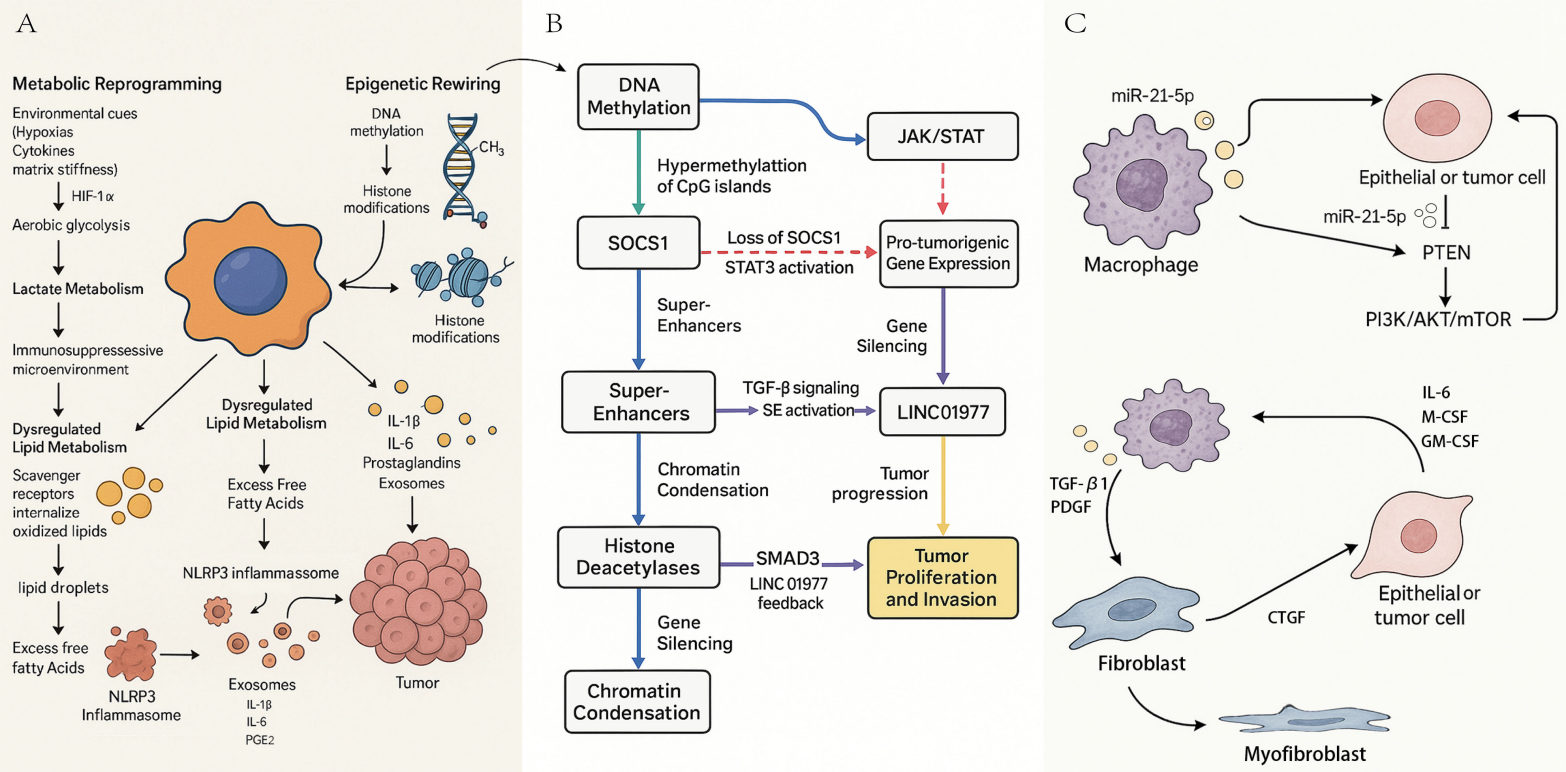
Regulatory networks underlying macrophage function in Pulmonary Fibrosis-Associated Lung Cancer (PF-LC). **(A)** Metabolic reprogramming of macrophages in the fibrotic and tumor microenvironment. HIF-1α, hypoxia inducible factor-1 alpha; IL-1β, interleukin-1 beta; IL-6, interleukin-6; NLRP3, NOD-like receptor family pyrin domain containing 3; PGE2, prostaglandin E2; **(B)** Epigenetic reprogramming and super-enhancer–driven transcriptional networks in macrophages. DNA, deoxyribonucleic acid; CpG, cytosine-phosphate-guanine; SOCS1, suppressor of cytokine signaling 1; JAK, Janus kinase; STAT, signal transducer and activator of transcription; STAT3, signal transducer and activator of transcription 3; TGF-β, transforming growth factor beta; SMAD3, SMAD family member 3; LINC01977, long intergenic non-protein coding RNA 1977; SE, super-enhancer; **(C)** Macrophages as communication hubs mediating multicellular signaling networks. miR-21-5p, microRNA-21-5p; PTEN, phosphatase and tensin homolog; PI3K, phosphoinositide 3-kinase; AKT, protein kinase B; mTOR, mechanistic target of rapamycin; IL-6, interleukin-6; M-CSF, macrophage colony-stimulating factor; GM-CSF, granulocyte-macrophage colony-stimulating factor; PDGF, platelet-derived growth factor; CTGF, connective tissue growth factor.

On the other hand, an excess of intracellular free fatty acids (particularly saturated fatty acids) strongly activates the NLRP3 inflammasome. This activation ultimately leads to the maturation and release of the potent proinflammatory cytokines IL-1β and IL-6 (172, 173). IL-1β further stimulates the expression of cyclooxygenase-2 (COX-2) and promotes the synthesis of prostaglandin E2 (PGE2) (174). Through its EP2 and EP4 receptors, PGE2 not only enhances the immunosuppressive function of macrophages, but also directly promotes the proliferation and invasion of cancer cells (175) (**Figure 3A**).

TREM2^+^ TAMs exhibit a distinct lipid-metabolic program, with increased fatty acid uptake, lipid droplet accumulation, and SPP1 expression, promoting immunosuppressive and pro-invasive phenotypes (176–178). MERTK^+^ macrophages show metabolic signatures consistent with efferocytosis-driven energy demand, while MARCO^+^ macrophages likely engage scavenger receptor–mediated lipid metabolism, broadly supporting M2-like functions (71).

### 3.2 Epigenetic reprogramming

Macrophages undergo functional reprogramming through epigenetic remodeling throughout the entire process of fibrosis progression to cancer (179, 180).

Cytokines prevalent in the fibrous microenvironment, such as TGF-β1, act as important primary factors in epigenetic reprogramming (181). These signals increase the activity of DNA methyltransferases (DNMTs) and thus induce abnormal hypermethylation of CpG islands in the promoter regions of important regulatory genes, ultimately leading to their transcriptional deactivation (182, 183). This deactivation mechanism primarily targets important negative regulators, such as cytokine suppression factor 1 (SOCS1) (184, 185). Epigenetic inactivation of SOCS1 disrupts the internal braking mechanism of the JAK/STAT signaling pathway, leading to permanent activation of the downstream transcription factor STAT3 (186). Once activated, STAT3 coordinates the regulation of gene expression of genes crucial for cell survival, proliferation, angiogenesis, and immunosuppression (e.g., VEGFA, PD-L1), thereby creating a microenvironment that favors tumor development (187, 188).

In PF-LC, histone modifications increase significantly (189, 190). Pro-inflammatory/pro-tumor macrophages show a marked increase in the activity of histone deacetylases (HDAC) – enzymes that forcibly induce chromatin condensation and gene suppression by removing acetyl groups from histones, particularly at the H3K18 position (189). This mechanism typically suppresses genes necessary for antitumor immunity, such as genes that regulate the interferon signaling pathway (191–193). Suppression of these pathways significantly impairs the antitumor activity of macrophages. It is important to note that DNA methylation and histone deacetylation often converge in the same regions of the genome, forming a dual mechanism that ensures stable maintenance of this immunosuppressive and pro-tumor state (194, 195).

Importantly, these epigenetic programs, though stable, are reversible: DNMT or HDAC inhibition can restore silenced antitumor genes and reprogram macrophages toward an immunostimulatory state (196, 197), while BET/BRD4 inhibition disrupts super-enhancer–driven pro-fibrotic and pro-tumor networks (198), highlighting the therapeutic potential of targeting these dynamic epigenetic states.

#### 3.2.1 Super-enhancer-driven core transcriptional networks

High-resolution chromatin access technologies (such as single-cell ATAC-seq) have identified a network of superactivators (SE) that are specifically activated in protumoral macrophages (199, 200). Superactivators are large regulatory domains consisting of dense clusters of potentiators that act as potent hubs, attracting high-density master transcription factors (e.g., STAT3, NF-κB) and key coactivators (particularly BET family proteins such as BRD4) (201). In the context of PF-LC, these SE-controlled gene networks carefully regulate the fundamental programs of macrophages that promote fibrosis, angiogenesis, and immunosuppression (189, 202). For example, TGF-β signaling in M2 macrophages activates SMD3, which binds to enhancers and stimulates expression of the long non-coding RNA LINC01977. This long RNA then forms a positive feedback loop with SMAD3, thereby enhancing tumor proliferation and invasive effects (202) (**Figure 3B**).

Targeting SE-associated regulators like BRD4 demonstrates that disrupting these hubs can reprogram macrophage states, indicating that SE-driven epigenetic programs are therapeutically accessible and not permanently fixed (198).

### 3.3 Macrophages as communication hubs: mediating multicellular signaling networks

Within the fibrosis-to-cancer continuum, macrophages function not merely as effector cells but as central communication hubs. Through soluble mediators and extracellular vesicles (EVs), they coordinate dynamic crosstalk with fibroblasts, epithelial cells, and tumor cells, thereby sustaining a self-reinforcing pathological signaling network (191–193).

#### 3.3.1 Extracellular vesicles

Macrophages associated with fibrosis are an important source of exosomes, especially microvesicles (191). These nanometer-sized vesicles carry a carefully selected cargo of biologically active molecules, including proteins, lipids, and non-coding RNA, such as microRNA (193). The exosome cargo is subsequently released into neighboring alveolar epithelial cells or emerging tumor cells, where it performs its biological functions (192). The transfer of miR-21-5p illustrates this mechanism (194, 195). Macrophages transport this microRNA to recipient cells via exosomes, allowing it to directly target the tumor suppressor gene PTEN and neutralize it (194). Inactivation of PTEN activates the PI3K/AKT/mTOR signaling pathway, a powerful driver of cell proliferation, survival, and EMT, which gives tumors invasive properties (199). The internal stability of exosomes in biological fluids makes their load very promising as a biomarker for liquid biopsies for the diagnosis and prognosis monitoring of PF-LC.

#### 3.3.2 Reciprocal crosstalk among macrophages, fibroblasts, and tumor cells

The progression of fibrosis to cancer is stimulated by a closely interconnected and self-reinforcing trilateral feedback loop involving macrophages, fibroblasts, and epithelial/cancer cells, leading to a continuous potentiation of pro-tumor signals (200–202). This cycle is usually initiated by activated macrophages, which secrete potent profibrotic factors such as TGF-β1 and PDGF (203). These signals have a strong impact on pulmonary fibroblasts, stimulating their differentiation into contractile and highly synthetic myofibroblasts (204, 205). These activated myofibroblasts then deposit a substantial extracellular matrix, which increases the biomechanical stiffness of the tissue, while simultaneously secreting pro-tumor factors such as CTGF (204). This biomechanical stiffness, combined with chemical signals, creates a microenvironment that promotes malignant proliferation and invasion. In response, tumor cells secrete cytokines such as M-CSF, GM-CSF, and IL-6. These factors attract additional monocyte precursors and enhance the profibrotic and immunosuppressive phenotype of macrophages, thus fueling a vicious cycle of progressive deterioration (206, 207) (**Figure 3C**).

## 4 Clinical application: implications for diagnosis and treatment

The progression of PF-LC is characterized by marked spatiotemporal heterogeneity, limiting the effectiveness of conventional analytical approaches and uniform treatment strategies. Elucidating macrophage dynamics across this continuum requires the integration of advanced technologies with patient-oriented precision diagnostic and therapeutic frameworks.

### 4.1 Spatiotemporal multi-omic

Spatial transcriptomics, particularly techniques now achieving subcellular resolution, are revolutionizing our understanding of the PF-LC microenvironment through *in situ* gene expression analysis within intact tissue architecturee (208–210). This technology transcends simple cell localization by precisely delineating the “pathogenic niche”—a functional ecosystem comprising pro-tumor macrophage subpopulations (e.g., SPP1+ macrophages) alongside fibroblasts and epithelial cells—thereby providing direct spatial evidence for the multi-cell synergistic mechanisms driving disease (211).

Importantly, from a diagnostic and translational perspective, spatial omics provides information that cannot be captured by bulk sequencing or dissociated single-cell approaches, namely the spatial confinement, persistence, and neighborhood interactions of pathogenic macrophage states within fibrotic and tumor-associated regions. Such spatial context enables identification of high-risk tissue architectures and immune-excluded niches, offering a framework for tissue-level risk stratification rather than population-level screening.

Furthermore, when combined with pseudo-time trajectory analysis, spatial omics technologies can directly deconstruct the temporal evolutionary trajectories of macrophages within their native tissue environment, tracing their pathogenic journey from pro-inflammatory states to pro-fibrotic states, and ultimately to pro-tumor states.

This spatial–temporal resolution supports the validation of disease-relevant macrophage programs and informs which molecular signals are most likely to yield clinically actionable circulating biomarkers.

Additionally, recent advances in high-content imaging and Cell Painting–based phenotypic profiling provide a complementary, morphology-centered approach to macrophage characterization, capturing reproducible changes in cell shape and organelle organization that may not be fully reflected at the transcriptomic level (212, 213). In PF–LC, this raises the possibility that macrophages recovered from bronchoalveolar lavage fluid (BALF) retain disease-imprinted morphological signatures associated with fibrotic remodeling and malignant transition. Although still exploratory, integrating Cell Painting with spatial omics and liquid biopsy frameworks may ultimately enable non-invasive inference of tissue-level pathogenic states through high-dimensional phenotypic profiling of lavage-derived macrophages.

#### 4.1.1 High-fidelity organoid models

To overcome the inherent limitations of two-dimensional culture, high-fidelity organoid co-culture systems have emerged. These systems, often integrated with organ-on-a-chip platforms, have become transformative tools (214). These models reproduce key features of the native lung, including its three-dimensional architecture, mechanotransduction induced by respiration, and precise hypoxic gradients characteristic of fibrotic and tumor tissues (215, 216). Researchers can precisely dissect complex cell-cell dialogues between macrophages and tumor cells and conduct high-throughput drug screening—with significantly greater *in vivo* translational relevance than traditional models (217). Although breakthroughs in chip vascularization technology have recently been achieved (218), he next phase of advancement lies in integrating migratory immune cells. By constructing “vascularized and immunoreactive” organoids featuring both perfused vascular networks and dynamic immune components, it will ultimately be possible to precisely recreate the entire process of tumor immune evasion, drug resistance, and immunotherapy response *in vitro*.

By enabling experimental perturbation of spatially defined macrophage–stromal interactions, these platforms provide functional validation of pathogenic niches first identified by spatial omics, thereby bridging discovery-phase diagnostics with therapeutic testing.

### 4.2 Future perspectives

The progression of PF-LC is characterized by marked spatiotemporal heterogeneity, limiting the effectiveness of conventional analytical approaches and uniform treatment strategies. Elucidating macrophage dynamics across this continuum requires the integration of advanced technologies with patient-oriented precision diagnostic and therapeutic frameworks.

#### 4.2.1 Precision patient stratification: from invasive biopsies to high-sensitivity liquid biopsies

Although single-cell omics technologies have identified numerous biomarkers associated with macrophages (such as SPP1 and MARCO) (52, 60), stratification of patients based on invasive tissue biopsies has proven to be clinically unjustified. In this sense, it is necessary to move towards BALF liquid biopsy, i.e., the analysis of circulating proteins or exosomes as non-invasive tools for the dynamic monitoring of patients (219).

Among these, SPP1 has emerged as a promising candidate for liquid biopsy. Macrophages that promote tumor growth continuously secrete SPP1, whose levels in circulating blood plasma strongly correlate with important clinical endpoints in lung adenocarcinoma, including poor prognosis, chemotherapy resistance, and lymph node metastasis (24). Consequently, this molecule acts as a powerful circulating surrogate marker for the activity of key pathological states of cells in the tumor microenvironment.

Similarly, the scavenger receptor MARCO, a new immune checkpoint expressed in macrophages that stimulate tumor growth, shows remarkable potential as a biomarker (73, 220, 221). In NSCLC and other solid tumors, MARCO identifies a distinct subpopulation of tumor-associated macrophages that is often associated with T-lymphocyte exclusion and PD-L1 coexpression (220, 221). Analyses of all cancer types confirm that MARCO expression is a strong negative prognostic indicator associated with the activation of important oncogenic signaling pathways such as TGF-β and PI3K/AKT/mTOR (222, 223). Therefore, the development of tests to detect circulating soluble MARCO is of critical importance. These tools provide non-invasive access to the immunological profile of the tumor, allowing both prediction of response to immunotherapy and recommendations for rational combination therapies combining anti-MARCO agents with existing checkpoint inhibitors.

#### 4.2.2 Targeted drug delivery

In addition to minimizing systemic toxicity, latest-generation therapies must ensure accurate drug delivery. Targeting the mannose receptor (CD206), which is highly expressed on the surface of tumor macrophages, has become a typical preclinical strategy (224). When modified with mannose ligands (Man-LNP), nanocarriers such as liposomes are effectively recognized and internalized by lung macrophages. This approach allows for a significant increase in the accumulation of the active substance at the tumor site and, at the same time, reduces its accumulation outside the target area in healthy organs (225).

These delivery platforms can be programmed to release their payload exclusively in specific organelles, such as acidic lysosomes, thereby maximizing therapeutic precision (226). Although a number of challenges remain before these high-tech nanotechnologies can be incorporated into everyday clinical practice, they undoubtedly represent the future of pulmonary fibrosis treatment. They promise to ultimately provide patients with safer and more effective treatment options with the potential for a cure.

#### 4.2.3 Macrophage-engineered immunotherapies

Recent advances in cell engineering have positioned macrophages as tractable therapeutic effectors in solid tumors, prompting growing interest in chimeric antigen receptor–engineered macrophages (CAR-macrophages, CAR-M) (227, 228). In contrast to CAR-T cells, macrophages display intrinsic tissue infiltration capacity, resilience to hypoxic and fibrotic microenvironments, and the ability to remodel extracellular matrix and antigen presentation, features that are particularly relevant in fibrosis-associated lung cancer niches (229, 230).

Notably, *in vivo* mRNA-LNP–based engineering of FAP-CAR macrophages has shown that macrophages can actively degrade fibrotic barriers, reduce collagen deposition, and restore drug and immune cell penetration in highly desmoplastic tumors, underscoring a functional advantage of CAR-macrophages over lymphocyte-based therapies in fibrotic tumor microenvironments (231). Recent *in vivo* studies using adoptive transfer or mRNA-LNP–based programming of CAR-macrophages have provided strong preclinical evidence for their safety, tissue engraftment, and antifibrotic efficacy, indicating that CAR-macrophage platforms are technically mature and adaptable for fibrotic microenvironments that are typically refractory to lymphocyte-based therapies (229, 232).

Together, these approaches position macrophage-centered cellular immunotherapies as a promising avenue for overcoming macrophage-driven immune suppression in PF–LC, particularly when integrated with precision biomarkers, spatial profiling, and targeted delivery systems.

### 4.3 Challenges and limitations

Despite advances in dissecting macrophage heterogeneity in the PF-LC transition, key challenges remain. Current organoid and spatial multi-omic models provide insights into 3D architecture and cell–cell interactions but are limited by incomplete systemic modeling, high cost, resolution constraints, and reliance on high-quality specimens. Many macrophage subsets identified by scRNA-seq may represent transient states, complicating selective targeting, while the stability and reversibility of epigenetic and metabolic programs remain uncertain. The integrative application of whole-genome sequencing and fragment-based methylation profiling offers a promising path to characterize these programs, but introduces computational and interpretive challenges. Addressing these gaps with multi-cellular, immune-responsive models and longitudinal multi-omic validation will be essential to translate mechanistic insights into clinical interventions.

## 5 Conclusion

PF-LC has a poor prognosis and represents a serious clinical problem, as progressive fibrosis often hinders effective surgical or oncological interventions, limiting therapeutic options in respiratory medicine. This review highlights the heterogeneity of macrophages as a key factor determining disease progression and focuses on how the SPP1+, MERTK+, TREM2+, and MARCO+ subpopulations synergistically create a profibrotic and immunosuppressive microenvironment through interactions with fibroblasts and tumor cells. Under the combined effects of matrix stiffness, hypoxia, and pulmonary dysbiosis, these macrophages undergo metabolic and epigenetic reprogramming through signaling pathways such as HIF-1α, TLR4-NF-κB, and super-enhancer-dependent transcription. From a clinical perspective, biomarkers derived from macrophages, such as SPP1 and MARCO, show promise for noninvasive diagnosis and risk stratification, while therapeutic approaches using targeted nanocarriers may mitigate treatment-limiting toxicity. Novel spatial multiomic technologies combined with advanced organoid models provide a translational framework for translating these discoveries into precise diagnostic and therapeutic strategies for the treatment of lung cancer associated with pulmonary fibrosis.
